# The Earliest European Hospital

**Published:** 1920-12-11

**Authors:** 


### The Earliest European Hospital.

At a meeting organised by the Pleasant Sunday Afternoon movement at Leamington, the Treasurer of Warneford Hospital, Sir J. L. Keir, K.C.B., was in the chair, and Mr. C. R. W. Offen gave an address on the origin of hospitals. He said that in pagan times hospitals were unknown, and the mental attitude towards the sick was either indolent indifference or active selfishness. Jerome records that the first hospital in Europe was opened by the noble patrician Christian Faviola in the third century, she herself ministering to their necessities, and even bearing the sick upon her back to her hospital when they were too feeble to walk there.

